# Special Issue: Anti-Inflammatory Activity of Plant Polyphenols 2.0

**DOI:** 10.3390/biomedicines10010037

**Published:** 2021-12-24

**Authors:** Enrico Sangiovanni, Mario Dell’Agli

**Affiliations:** Department of Pharmacological and Biomolecular Sciences, Università degli Studi di Milano, Via Balzaretti 9, 20133 Milano, Italy; mario.dellagli@unimi.it

## 1. Introduction

Inflammation is a complex process that occurs in response to infections or other tissue damages, such as trauma, wounds, burns, and toxic substances. The inflammatory reaction is part of the human immune defense system and, macroscopically, can be characterized by five typical symptoms, mainly caused by local vascular changes and leukocyte activation: heat, redness, pain, swelling, and temporary loss of function. Overall, this fundamental process aims to protect the human body by avoiding the spread of harmful agents and to restore tissues and cellular homeostasis. However, when the causative agent is not properly removed, a chronic inflammatory state can occur.

Over time, the chronic stress causes damages ranging from loss of tissue function to DNA mutations, which can promote cancer cell formation. In this progression of damage, the continuous production of reactive oxygen species (ROS) plays a determining role. Oxidative stress is described as an altered equilibrium between the generation of ROS and the endogenous antioxidant defenses.

The plant kingdom remains an important resource of molecules with therapeutic potential for humans. Several plants have been shown to possess anti-inflammatory activities in clinical studies owing to the presence of secondary metabolites [1,2]. Polyphenols can directly act against pathogens such as viruses and bacteria, thus indirectly resolving the inflammatory process, as extensively demonstrated in propolis [3,4].

Nuclear factor κB (NF-κB) is a key transcription factor responsible for the expression of cytokines, chemokines, and other cellular inflammatory mediators and is considered one of the main proinflammatory pathways [5]. In physiological conditions, this factor is found in an inactive form in the cytoplasm, bound to its own inhibitory protein IκBα. However, the presence of proinflammatory stimuli, such as infections, ROS, or other endogenous proinflammatory mediators, leads to the rapid activation of NF-κB, which translocates into the nucleus. NF-κB is an excellent target for evaluating the anti-inflammatory activity of various molecules, including those of plant origin. In fact, several classes of polyphenols can inhibit the action of this transcription factor in different cellular models [6,7,8].

Polyphenols are molecules with excellent antioxidant capacity owing to the presence of phenolic rings, which act as electron traps for ROS. Despite this direct action, the most recent scientific evidence has shown that the best antioxidant activity is obtained by the stimulation of endogenous antioxidant defenses.

Nuclear factor erythroid 2–related factor 2 (Nrf2) regulates the expression of endogenous antioxidant enzymes, such as catalase and superoxide dismutase. In resting conditions, this factor is bound to its inhibitory protein, the kelch-like ECH associated protein (Keap1). Nrf2 is activated by increased oxidative stress, which induces the detachment from Keap1 and the binding of Nrf2 to the antioxidant element (ARE), hence promoting the transcription of cytoprotective enzymes [9].

Oxidative stress plays a dual role in inflamed cells; on the one hand, it is a fundamental factor for signal transmission for immunomodulation and apoptosis, but on the other hand, it is a potential source of DNA mutations, capable of inducing DNA strand lesions and nitration of the guanine bases. Between the several genes implicated in DNA damages and repair response, the tumor suppressor p53 gene has a key role, acting as a transcription regulator under stress conditions.

The NF-κB, Nrf2, and p53 pathways are deeply connected, and their regulation can change the cellular fate. For example, p53 acts as a suppressor of inflammation, and it can inhibit the transcriptional activity of NF-κB [10].

The purpose of this Special Issue is to contribute to the collection of scientific evidence on the anti-inflammatory properties of polyphenols, focusing on the role of individual compounds and their respective mechanisms of action, including anti-inflammatory and antioxidant effects.

## 2. Recent Advances in the Role of Polyphenols in Inflammatory Conditions

In this Special Issue, scientific papers concerning different pathological contexts are collected, from renal function to antiviral properties, but also cancer prevention. The studies include both in vitro and in vivo effects and describe bioavailability data of active principles, when available. This collection focuses mainly on pure compounds and the mechanisms of action underlying their anti-inflammatory and antioxidant activities.

Galli A. et al. [11] investigated the effect of verbascoside on pancreatic β-cells. This phenylethanoid glycoside is present in the wastewater of olive fruit processing. Verbascoside can be extracted from *Olea europea*, but also from other plant families. The authors demonstrated that verbascoside prevents β-cells’ oxidative stress by decreasing ROS content and lipid peroxidation through the activation of the Nrf2 pathway. In addition, verbascoside reduced the activation of the NF-κB pathway, protecting pancreatic β-cells from death, but also preserved the mitochondrial membrane potential under basal and stressed conditions. These effects were obtained by treating cells with a concentration of 16 µM of verbascoside. The results of this study set the basis for the possible use of verbascoside in protecting β-cells from oxidative stress and inflammation, conditions involved in type 2 diabetes progression.

Curcumin is a well-known curcuminoid extracted from the rhizome of *Curcuma longa*. Various biological activities of curcumin have been described in the literature including antioxidant, antimicrobial, and anti-inflammatory properties [12]. Zoi V. et al. [13] explored the possible use of curcumin as a radio-sensitizing agent in the treatment of glioblastoma, the most common and severe malignant brain tumor. The authors discovered that curcumin (3–26 µM) improves the radiosensitive status of glioblastoma cancer cells (U87 and T98, exposed to 2 Gy or 4 Gy of irradiation), resulting in a higher inhibitory effect compared to radiation or curcumin alone. The mechanism of action involved G2/M arrest in the cell cycle. Furthermore, curcumin showed synergistic effects even when combined with the chemotherapeutic agent temozolomide. Other researchers have positively described the association of curcumin with other chemotherapeutic agents [14] and the use of liposomal formulations [15]. These findings encourage further studies to evaluate the potential use of curcumin alongside radiotherapy as an innovative strategy for the treatment of glioblastoma.

Vereen B. et al. evaluated the anti-renal fibrosis effect of an extract from *Antirhea borbonica*, a French medicinal plant found in Reunion Island, in comparison to caffeic acid, one of the major phenolic acids of the extract, in mice subjected to unilateral ureteral obstruction [16]. Mice were treated daily with an oral dose of 25 mg/kg of *Antirhea borbonica* or 25 mg/kg of caffeic acid by gavage. Both the extract and caffeic acid reduced macrophage infiltration and induced a down-regulation of pro-inflammatory and pro-fibrotic cytokines (Tgf-β, Tnf-α), chemokines (Mcp1), and inhibition of NF-κB. Polyphenols from *Antirhea borbonica* also significantly increased Nrf2 mRNA expression and subsequent CAT and Cu/ZnSOD enzymes. The authors concluded that the in vivo nephroprotective effects of *Antirhea borbonica* is partially supported by the presence of caffeic acid, but the contribution of other polyphenols present in the plant extract cannot be excluded.

Siddiqui S.S. et al. reviewed the effects of the main dietary flavonoids focusing on their ability to prevent inflammation and cancer in relation to p53-mediated mechanisms [17]. The flavonoids explored by the authors were quercetin, luteolin, cyanidin, daidzein, and epigallocatechin gallate (EGCG). This group of flavonoids exert their anti-inflammatory effects mainly by inhibiting the Janus kinase-signal transducer and activator of transcription (JAK-STAT), NF-κB, and mitogen-activated protein kinase (MAPK) pathways. The effects involved a reduction in cytokines (IL-1β, TNF-α, IL-6, and IL-8) and cell death markers (caspase-3). The authors concluded that these flavonoids could reduce inflammation and ultimately lead to cancer prevention, thus emphasizing the need for further studies to explore the connections between the p53 and Nrf2 pathways.

Finally, the study of Besdnova N.N. considered the antiviral effects of polyphenols from marine algae, reviewing the biological activities and their mechanism of actions [18]. Macro- and microalgae can accumulate phloroglucinol and related polymers, named phlorotannins. Phlorotannins are a class of heterogenous compounds in terms of molecular weight and level of isomerization; some of these structures can be sulphated or halogenated. Seaweed phlorotannins are composed of eight phenolic rings, which give a greater antioxidant power than that of tannins produced by terrestrial plants, which possess only three or four phenolic rings. The authors highlighted the ability of phlorotannins to decrease viral load, acting at different stages of the viral cell cycle by interfering, for example, with the attachment to the cell surface, exerting a direct antiviral effect or blocking viral enzymes. Concomitantly, phlorotannins can enhance antioxidant defense and reduce inflammation by lowering the production of proinflammatory cytokines and inflammatory cell migration.

## 3. Conclusions and Future Perspectives

The data collected in this Special Issue once again confirm the importance of the plant kingdom for the search for new compounds with anti-inflammatory activity. Inflammation plays a central role in various pathological contexts, ranging from acute infection to metabolic or chronic damages. The scientific literature underlines the close correlation between inflammatory markers, ROS, and DNA damage; the latter is capable of increasing the probability of tumor formations.

In this context, polyphenols can play a fundamental role both in curative and preventive terms, also due to their dietary intake. Despite the large body of preclinical literature, there is still a lack of clinical data on polyphenols that can confirm their activities in humans, especially in the case of extracts intended as mixtures of different components. The main limitations are due to the variability of the composition of the plant matrices and the lack of titration in active ingredients. However, these studies confirm that even isolated polyphenols can have interesting biological activities.

Although polyphenols have shown various benefits in the inflammatory field, one of the main limitations for their use in humans concerns their bioavailability. In this regard, various strategies are being studied to improve the absorption of these compounds, and the use of phytosomal formulations appears to be one of the most promising ways [19].
